# Identification of a Glycolysis-Related LncRNA Signature to Predict Survival in Diffuse Glioma Patients

**DOI:** 10.3389/fonc.2020.597877

**Published:** 2021-02-05

**Authors:** Yangyang Wang, Wenjianlong Zhou, Shunchang Ma, Xiudong Guan, Dainan Zhang, Jiayi Peng, Xi Wang, Linhao Yuan, Peiliang Li, Beibei Mao, Peng Kang, Deling Li, Chuanbao Zhang, Wang Jia

**Affiliations:** ^1^ Department of Neurosurgery, Beijing Tiantan Hospital, Capital Medical University, Beijing, China; ^2^ Beijing Neurosurgery Research Institute, Capital Medical University, Beijing, China; ^3^ China National Clinical Research Center for Neurological Diseases (NCRC-ND), Beijing, China

**Keywords:** glioma, glycolysis, long non-coding RNAs (LncRNA), prognosis, risk model

## Abstract

Glycolysis refers to one of the critical phenotypes of tumor cells, regulating tumor cell phenotypes and generating sufficient energy for glioma cells. A range of noticeable genes [such as isocitrate dehydrogenase (IDH), phosphatase, and tensin homolog (PTEN), or Ras] overall impact cell proliferation, invasion, cell cycle, and metastasis through glycolysis. Moreover, long non-coding RNAs (LncRNAs) are increasingly critical to disease progression. Accordingly, this study aimed to identify whether glycolysis-related LncRNAs have potential prognostic value for glioma patients. First, co-expression network between glycolysis-related protein-coding RNAs and LncRNAs was established according to Pearson correlation (Filter: |r| > 0.5 & P < 0.001). Furthermore, based on univariate Cox regression, the Least Absolute Shrinkage and Selection Operator (LASSO) analysis and multivariate Cox regression, a predictive model were built; vital glycolysis-related LncRNAs were identified; the risk score of every single patient was calculated. Moreover, receiver operating characteristic (ROC) curve analysis, gene set enrichment analysis (GSEA), GO and KEGG enrichment analysis were performed to assess the effect of risk score among glioma patients. 685 cases (including RNA sequences and clinical information) from two different cohorts of the Chinese Glioma Genome Atlas (CGGA) database were acquired. Based on the mentioned methods, the risk score calculation formula was yielded as follows: Risk score = (0.19 × EXP_FOXD2-AS1_) + (−0.27 × EXP_AC062021.1_) + (−0.16 × EXP_AF131216.5_) + (−0.05 × EXP_LINC00844_) + (0.11 × EXP_CRNDE_) + (0.35 × EXP_LINC00665_). The risk score was independently related to prognosis, and every single mentioned LncRNAs was significantly related to the overall survival of patients. Moreover, functional enrichment analysis indicated that the biologic process of the high-risk score was mainly involved in the cell cycle and DNA replication signaling pathway. This study confirmed that glycolysis-related LncRNAs significantly impact poor prognosis and short overall survival and may act as therapeutic targets in the future.

## Introduction

Glioma refers to the most frequent and malignant primary central nervous system (CNS) tumor happening to adults, exhibiting a high recurrence rate, high morbidity, and mortality (1). Although administrated with comprehensive therapy (such as surgical resection, radiotherapy, and chemotherapy), patients with glioma exhibit extremely poor prognosis (2). Over the past few years, a wide variety of biomarkers have been reported to facilitate the diagnosis and prognosis in various cancers. In 2016, the World Health Organization (WHO) initially revised the classification of the CNS tumors depending on morphology and molecular parameters, suggesting that molecular testing has been of critical significance to the diagnosis of glioma (3). Even though the progress of molecular signatures has led to remarkable advancement of diagnosis and prognosis, it remains unsatisfactory and appears much room for improvement (4). Therefore, more and better fit molecular models are urgently required.

It has been increasingly evidenced that long non-coding RNAs (LncRNAs) are vital to regulating signal pathways and disease progression [*e.g.*, well-known CRNDE (5, 6), MALAT1 (7), H19 (8)]. Meanwhile, in contrast to normal cells, glycolysis is one of the critical phenotypes of tumor cells, contributing to altering tumor cell phenotypes and generating sufficient energy for tumor needs. Existing studies reported that glycolysis overall impacts tumor cell growth, proliferation, migration, and distant metastasis (9–11). However, what effect glycolysis-related LncRNAs exerts on glioma patients remains unclear, as well as whether glycolysis-related LncRNAs have potential value in the diagnosis of glioma and serve as potential therapeutic targets or not remains unexplored.

In the present study, we screened the glycolysis-related LncRNAs in glioma patients based on the RNA sequences (RNAseq) data of the Chinese Glioma Genome Atlas (CGGA). According to Cox regression and the Least Absolute Shrinkage and Selection Operator (LASSO) regression analysis, patients can be split into two groups based on the risk score: low- and high-risk groups. Furthermore, the differences between the two groups were assessed from multiple perspectives. Overall, the identified signature of glycolysis-related LncRNAs was demonstrated as an independent prognostic factor and a potential therapeutic strategy for glioma patients in both of the two cohorts.

## Materials and Methods

### Patients and Datasets

**Figure 1:**
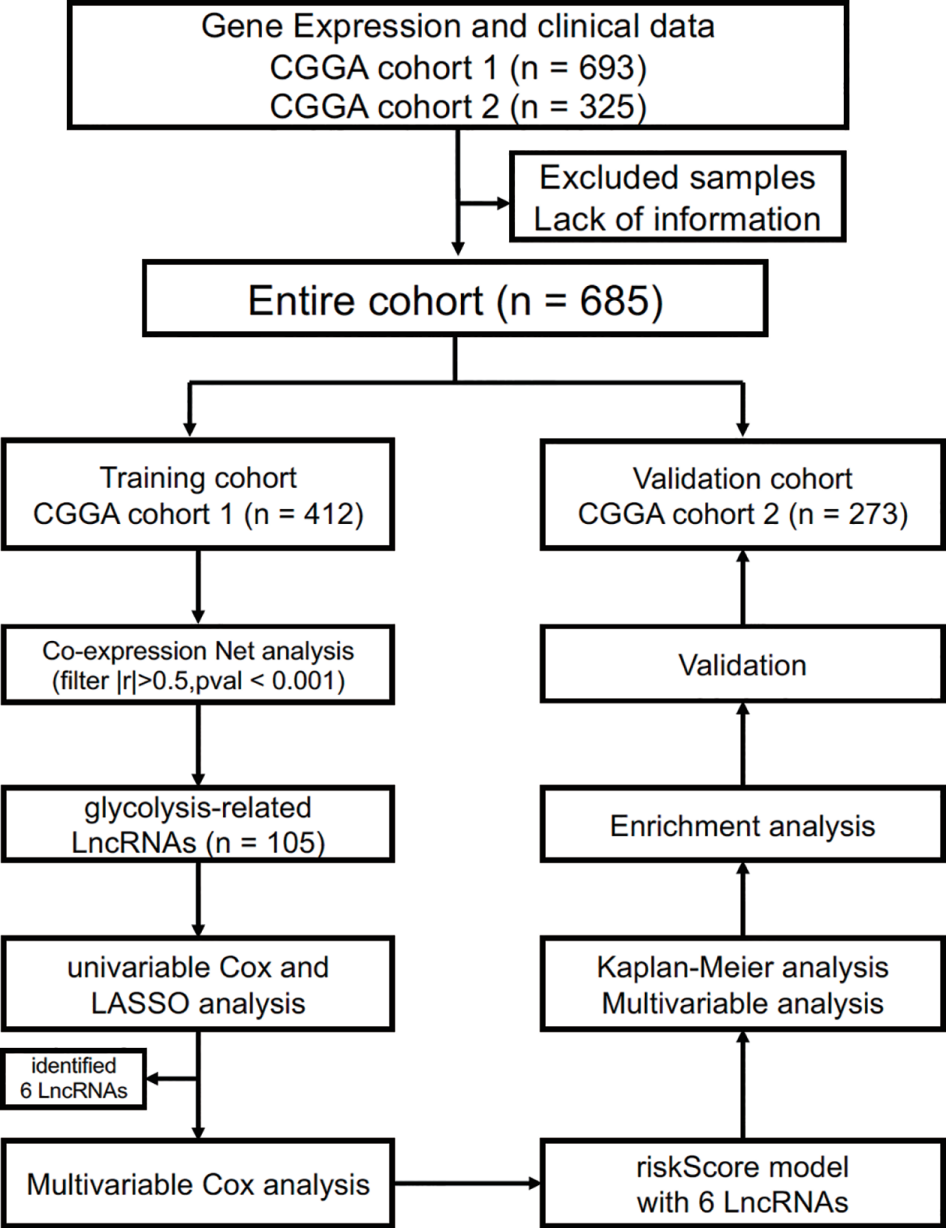
Flow chart of data collection and analysis.

The present study was approved by the Institutional Review Board of the Beijing Tiantan Hospital affiliated with Capital Medical University. In the training cohort, RNAseq data and clinical information (such as age, gender, tumor grade, molecular mutation and follow-up data) were acquired from the CGGA database (http://www.cgga.org.cn/) (12, 13). Likewise, the validation cohort was obtained from another glioma cohort of the CGGA database (14, 15). Gene expression was normalized and calculated using the RPKM (reads per kilobase transcriptome per million reads) method (16). Therefore, we could directly use the RPKM values for subsequent analysis. We defined the survival as overall survival (OS), which was calculated from the date of initial diagnosis to the date of death or last follow-up. The process of this study is presented in **Figure 1**.

### Glycolysis-Related Long Non-Coding RNA Extraction

First, in accordance with Hg19 RefSeq (RNA sequences, GRCh37) downloaded from the UCSC Genome Browser (http://genome.ucsc.edu/), genes were classified into protein-coding and long non-coding genes. Four glycolysis-related gene sets (Hallmark glycolysis, KEGG glycolysis gluconeogenesis, Biocarta glycolysis pathway, and Reactome glycolysis) were acquired from the Gene Set Enrichment Analysis (GSEA) database (http://www.broad.mit.edu/gsea/msigdb/) (17). Subsequently, to build glycolysis-related LncRNA co-expression networks with glycolysis-related genes, the Pearson correlation between them was determined with the R package “corrplot”. Lastly, 105 glycolysis-related LncRNAs were identified in the training cohort. (Filter: |r| > 0.5 and P < 0.001).

### Building of the Predictive Model by Cox Regression and Least Absolute Shrinkage And Selection Operator Analysis

The univariate Cox analysis was conducted with R package “survival” to primarily screen out glycolysis-related LncRNAs with prognostic value based on the P values (P < 0.001). Considering correlation and collinearity between variables, the RPKM values of the LncRNAs screened out by the univariate Cox analysis were subsequently included in the LASSO regression with R package “glmnet”. The 1-standard error of the minimum criteria was used to tune the regularization parameter (*λ*), and maxit was set to 1,000. Furthermore, the selected glycolysis-related LncRNAs with non-zero coefficients were employed in a multivariate Cox regression model using the enter method to calculate the risk score. The risk score formula is written as:

$$Risk\;score=\sum_{i=1}^{n}coefficient\left({lncRNA}_{i}\right)\times expr\left({lncRNA}_{i}\right)$$

### Enrichment Analysis

GSEA was performed to identify different biological processes between the low- and high-risk groups with the GSEA v7.1 software (http://www.broadinstitute.org/gsea/) (17). Correlation analysis was conducted between risk score and protein-coding RNAs. After that, enrichment analysis, including GO analysis and KEGG analysis, was applied for the correlated genes (|r| > 0.4, P < 0.05). The analysis was carried out using the package “clusterProfiler” of R project (18). P <0.05 was regarded as a significant outcome.

### Statistical Analysis

Normal distributions for continuous variables were assessed by the Shapiro–Wilk tests. Kaplan–Meier method was employed to evaluate survival with the Log-rank test to assess the differences between the mentioned two subgroups. Independent prognostic variables were adopted for survival assessment with Cox and LASSO regression models. Receiver operating characteristic (ROC) curve and area under the curve (AUC) at 3- and 5-year were calculated to assess the predictive performance of risk score with the “timeROC” package. R (version 3.6.3), and its packages were adopted for the analysis of all data (https://www.r-project.org/). Two-tailed p-value was adopted, and P <0.05 was considered to be statistically significant.

## Results

### Pre-Processing of Patient Data

Overall, 685 glioma cases were collected from the CGGA database after removing missing values; the information of 412 glioma cases (such as RNAseq data and clinical features) was used as the training cohort, and 273 of them, collected from another glioma cohort of the CGGA database, were used to evaluate the identified signature performance. Specific sample information regarding clinical characteristics is listed in **Table 1**.

**Table 1:** Clinical characteristics of diffuse glioma patients.

Characteristic	Training cohort	Validation cohort	P value
	(n = 412)	(n = 273)	
Age			
≤45	247 (60.0%)	164 (60.1%)	0.97
>45	165 (40.0%)	109 (39.9%)	
Gender			
Female	181 (43.9%)	105 (38.5%)	0.16
Male	231 (36.1%)	168 (61.5%)	
Grade			
LGG	257 (62.4%)	146 (53.5%)	0.02
GBM	155 (37.6%)	127 (46.5%)	
IDH mutation			
No	185 (44.9%)	130 (47.6%)	0.48
Yes	227 (55.1%)	143 (52.4%)	
1p19q codeletion			
No	325 (78.9%)	219 (80.2%)	0.67
Yes	87 (21.1%)	54 (19.8%)	
MGMT			
Unmethylated	168 (40.8%)	131 (48.0%)	0.06
Methylated	244 (59.2%)	142 (52.0%)	
TMZ			
No	88 (21.4%)	96 (35.2%)	<0.001
Yes	324 (78.6%)	177 (64.8%)	
Radiation therapy			
No	84 (20.4%)	57 (20.9%)	0.88
Yes	328 (79.6)	216 (79.1%)	

P value based on Chi-square test (binary variables).

### Cox Regression and Least Absolute Shrinkage and Selection Operator Analysis

**Figure 2:**
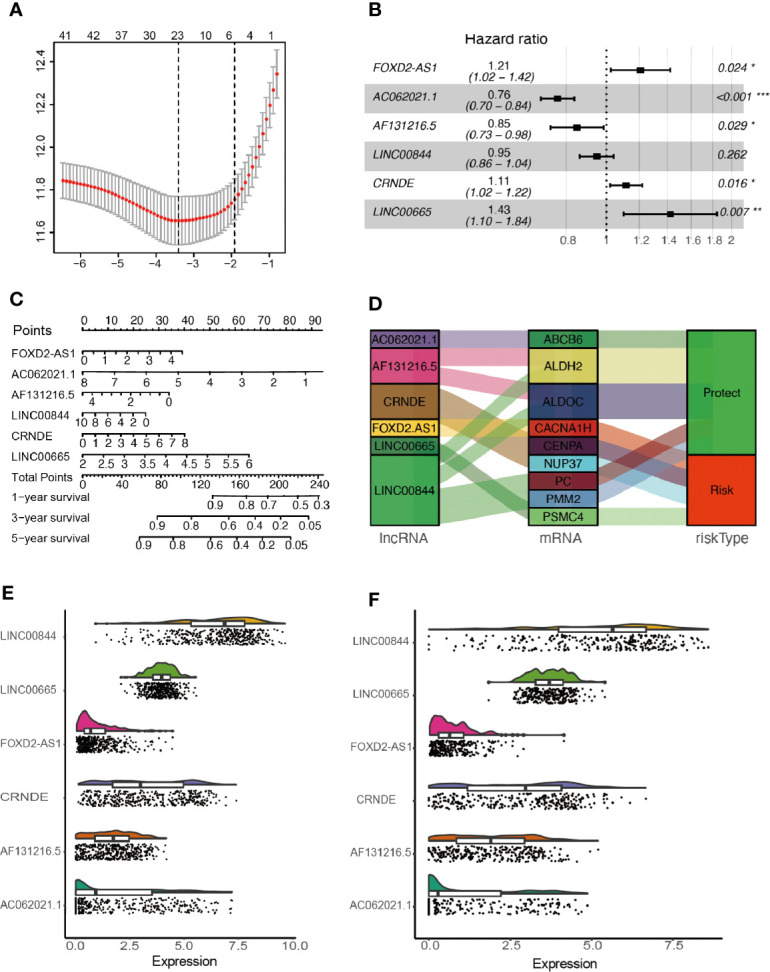
Network construction of the co-expressed and identification of key LncRNAs. **(A)** Texture feature selection using the LASSO regression model, *λ* value was chosen (1-SE criteria) according to cross-validation, where optimal *λ* resulted in six non-zero coefficients in the training cohort. **(B)** Construction of a risk signature to predict glioma prognosis by multivariate Cox regression. **(C)** Nomogram to predict the 1-, 3-, 5-year OS. **(D)** Sankey diagrams depicting the relationship of glycolysis-related protein-coding genes and glycolysis-related LncRNAs. **(E, F)** Expression of identified glycolysis-related LncRNAs in the training and validation cohorts. *P < 0.05, **P < 0.01, and ***P < 0.001.

After removing overlapped genes, a glycolysis-related set was generated, covering 298 genes from the Molecular Signatures Database. 402 LncRNAs were identified according to Hg19 RefSeq (RNA sequences, GRCh37). By conducting the correlation analysis between 298 glycolysis-related protein-coding genes and LncRNAs, 105 glycolysis-related LncRNAs were screened out in the training cohort (Filter: |r| > 0.5 and P < 0.001). Furthermore, 42 survival-related LncRNAs were identified with univariate Cox analysis in the training cohort. 42 variables were reduced to six potential predictors in the training cohort (7:1 ratio) by using LASSO analysis (**Figure 2A**). In addition, the features with non-zero coefficients were employed in a multivariate Cox regression model to calculate the risk score of both the training and validation cohorts (**Figures 2B–D**). The expression of identified LncRNAs in both training and validation cohorts is illustrated in **Figures 2E, F**.

The mentioned LncRNAs were expressed in the calculation formula.

$$\text{Risk score}=\left(0.19\times {\text{EXP}}_{\text{FOXD}2-\text{AS}1}\right)+\left(-0.27\times {\text{EXP}}_{\text{AC}062021.1}\right)+\left(-0.16\times {\text{EXP}}_{\text{AF}131216.5}\right)+\left(-0.05\times {\text{EXP}}_{\text{LINC}00844}\right)+\left(0.11\times {\text{EXP}}_{\text{CRNDE}}\right)+\left(0.35\times \text{EXP}\text{LINC}00665\right)$$

### Association Between Risk Score and Patient Outcome

**Figure 3:**
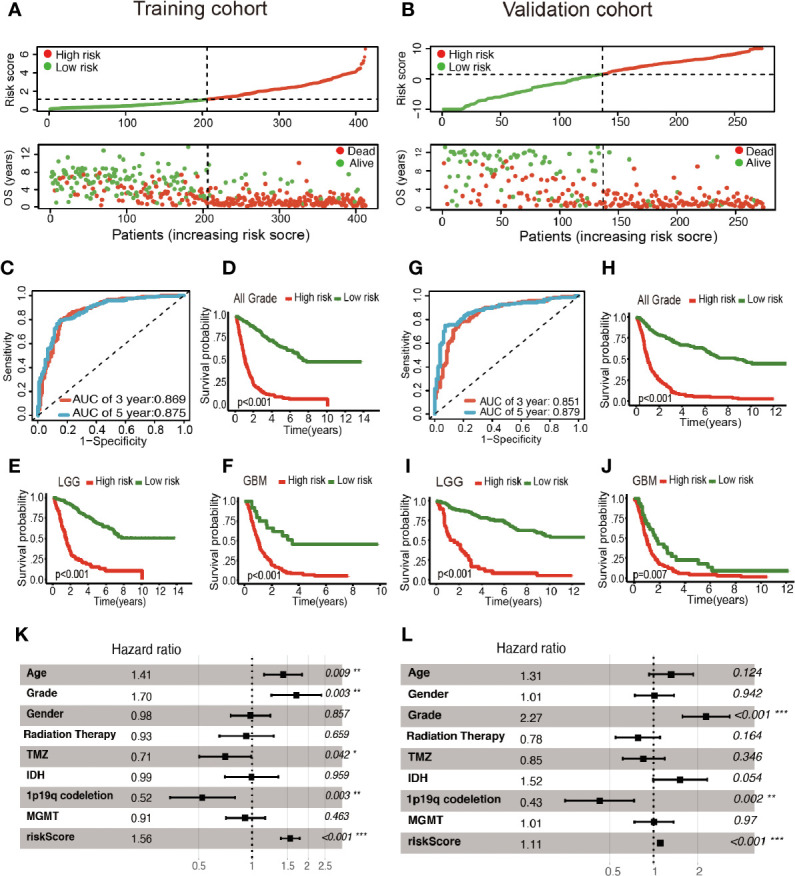
Construction of the glycolysis-related LncRNAs signature for survival prediction. **(A, B)** Risk score distribution, survival status for patients in low- and high-risk groups by the LncRNA signature. ROC curves of the signature for predicting 3- and 5- year survival of glioma in both training **(C)** and validation cohorts **(G)**. Kaplan**–**Meier curve based on the identified survival-related glycolysis LncRNAs in the training **(D–F)** and validation **(H–J)** cohorts. **(K, L)** Multivariable comparison of clinical features and the risk score. *P < 0.05, **P < 0.01, and ***P < 0.001.

Patients were classified into two cohorts in accordance with the risk score median value. Besides, mortality was elevated with the increase in risk score (**Figures 3A, B** **)**. Furthermore, Kaplan–Meier and ROC curve were plotted to subsequently measure the effect of the identified glycolysis-related LncRNAs. In the training cohort, AUC was 0.869 in the 3rd year and 0.875 in the 5th year in the training cohort (**Figure 3C**); Kaplan−Meier analysis also indicated that the risk score based on glycolysis-related LncRNAs could act as an effective prognostic indicator for glioma patients (**Figures 3D–F**). In the validation cohort, the AUC was 0.851 and 0.879 in the 3rd year and the 5th year, respectively (**Figure 3G**). Kaplan–Meier analysis of the validation cohort was the same as the training cohort (**Figures 3H–J**). The multifactor analysis demonstrated that the risk score was independently associated with OS in the training and validation cohorts. The significant relationships between survival and factors in the training cohort were observed as follows: risk score (High *vs*. Low) (hazard ratio (HR): 1.56; 95% confidence interval (CI): 1.37–1.77; P < 0.001), age (>45yrs *vs*. ≤45yrs) (HR: 1.41; 95% CI: 1.09–1.83; P = 0.009), grade (GBM *vs*. LGG) (HR: 1.70; 95% CI: 1.20–2.39; P = 0.003), TMZ (Yes *vs*. No) (HR: 0.71; 95% CI: 0.50–0.99; P = 0.042), and 1p19q codeletion (Yes *vs*. No) (HR: 0.52; 95% CI: 0.34–0.80; P = 0.003) exhibited significant associations with OS. The identical method was applied in the validation cohort, and the following results significantly associated with OS were indicated: risk score (High *vs*. Low) (HR: 1.11; 95% CI: 1.07–1.15; P < 0.001), Grade (GBM *vs*. LGG) (HR: 2.27; 95% CI: 1.57–3.29; P < 0.001) and 1p19q codeletion (Yes *vs*. No) (HR: 0.43; 95% CI: 0.25–0.74; P = 0.002). As revealed from the mentioned results, the risk score was of significance in the prognosis and OS prediction of glioma patients in the multivariate analysis with P <0.001 in both training and validation cohorts (**Figures 3K, L** **)**.

### Mentioned Long Non-Coding RNAs Were Associated With Clinicopathological Characteristics and Can Predict Survival Status in Gliomas

**Figure 4:**
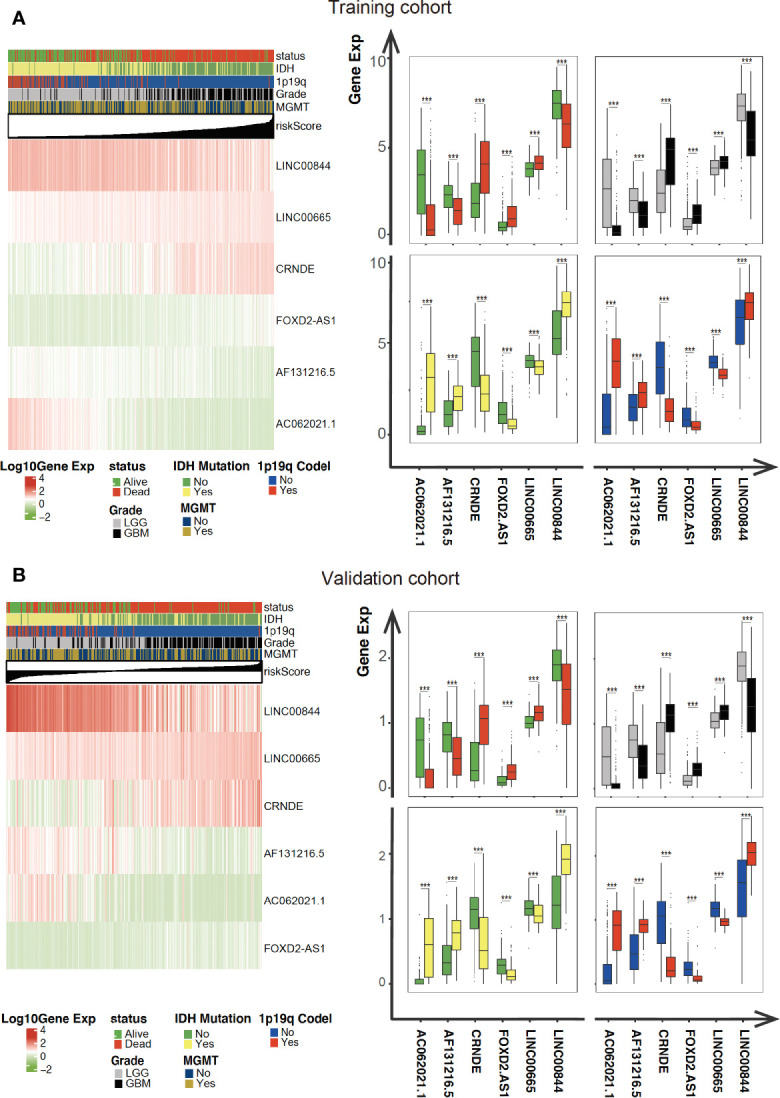
Expression of the mentioned glycolysis-related LncRNAs is correlated with clinicopathological features and prognosis of gliomas. **(A, B)** Heatmaps showing the expression levels of six LncRNAs in gliomas with different groups. ***P < 0.001.

**Figure 5:**
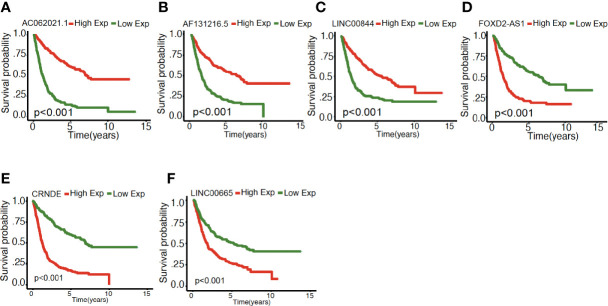
**(A–F)** Mentioned gene in Kaplan**–**Meier analysis in the training cohort.

As illustrated in **Figure 4**, the association between the six identified LncRNAs and the pathological characteristics (including survival status, survival time, WHO grade, IDH mutation status, 1p/19q codeletion status, and MGMT status) was analyzed. LncRNAs expression and pathological characteristics are presented as heatmaps and boxplots (**Figures 4A, B** **)**, suggesting that the expression of every single LncRNAs was significantly different in different groups. Kaplan–Meier analysis demonstrated that each of the mentioned LncRNAs was significantly related to survival status in glioma in both training and validation cohorts (**Figures 5A–F** and **S1A–F**).

### Enrichment Analysis

**Figure 6:**
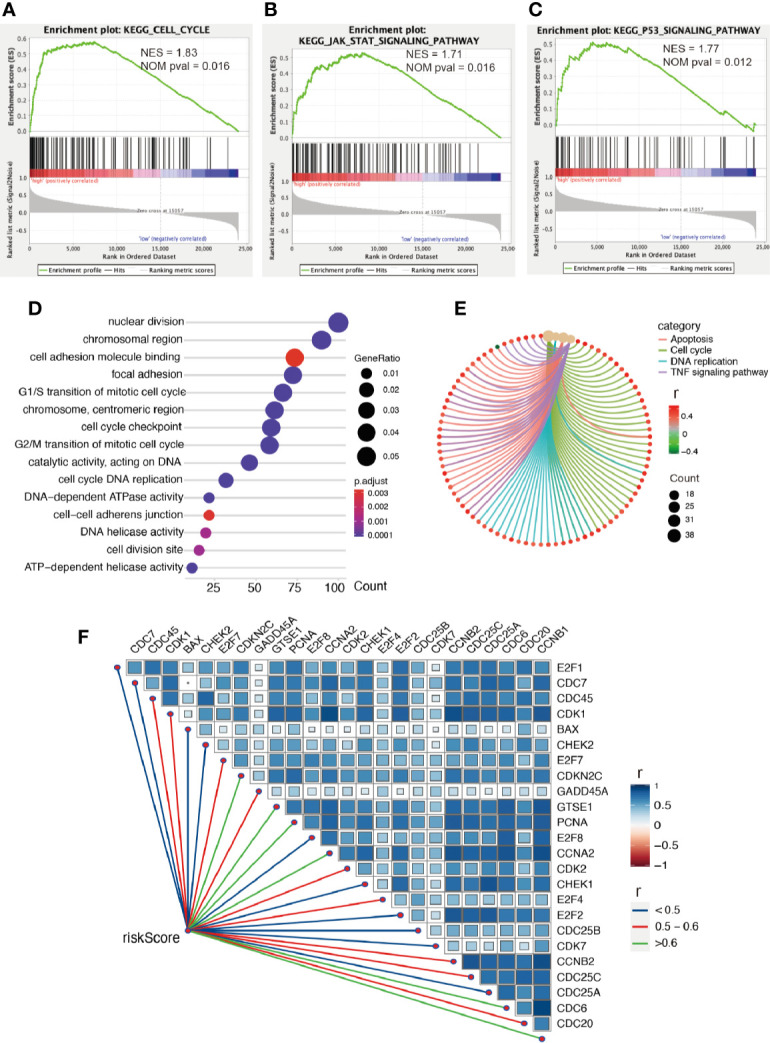
Enrichment analysis. **(A–C)** GSEA between low- and high-risk groups revealing cell cycle and signaling pathway regulating cell proliferation. Functional enrichment analysis including GO **(D)** and KEGG **(E)** indicting the biological process risk score was mainly involved in cell proliferation. **(F)** The association between risk score and genes regulating cell cycle, all P values < 0.001.

Firstly, we conducted GESA to further clarify biological processes related to the risk score. Cell cycle, JAK-STAT signaling pathway, and P53 signaling pathway were significantly enriched between the two groups relying on risk score in the training cohort (**Figures 6A–C**). Cell cycle, P53 signaling pathway, and apoptosis were enriched in the validation cohort (**Figures S2A–C**). Meanwhile, the 1875 genes in the training cohort and 2620 genes in the validation cohort were analyzed by enrichment analysis, and they were strongly associated with risk score by Pearson correlation analysis (Pearson |r| > 0.4, P < 0.05). As illustrated in **Figures 6D, E**, enrichment analysis indicated that GO was mainly enriched in cell division site, cell cycle, cell proliferation, cell–cell adheres junction, and DNA replication, while KEGG was mainly enriched in cell cycle, apoptosis, and TNF signaling pathway. In the validation cohort, similar outcomes were obtained (**Figures S2D, E**). Additionally, correlation analysis showed risk score was strongly related to the genes regulating cell cycle, as illustrated in **Figures 6F** and **S2F**.

## Discussion

Glioma is considered the most frequent brain tumor that jeopardizes human health and causes more years of life lost for its malignant progression and poor prognosis, especially glioblastoma. Even with the most comprehensive treatment (such as surgery, radiation, chemotherapy and immunotherapy), the existing therapeutic effects on glioma have been unsatisfactory (19). Although great strides have been achieved over the past few years in advancing the treatment effect, as impacted by residence to various treatments, it is difficult to cure completely, and patients are susceptible to recurrence after the first treatment, causing poor clinical prognosis among glioma patients (20–22). Thus, a series of novel biomarkers should be discovered as a potential therapeutic target to improve prognosis.

Glycolysis rate is 100 times higher in tumor cells compared to the normal cells (23, 24). Thus, the difference between them has aroused huge attention from researchers in recent years. The differences in glycolysis lie in various WHO grades and exhibit a high prognostic value in glioma patients (25). Numerous studies reported that some well-known biomarkers (such as IDH 1 or 2, PTEN, and Ras) can affect glycolysis, which is associated with poor prognosis. For instance, Lu et al. demonstrated that the protein PTEN, a well-known biomarker regulating survival, proliferation, and cell growth, is significantly associated with glycolysis through PGK1 in brain tumors (26). Kloog et al. reported that Ras inhibition induces cell death through glycolysis shutdown in glioblastoma (27). Zhou et al. showed that compared to the IDH1 wild-type, the intermediate products of glycolysis are significantly reduced in the glioma tissues with IDH1 mutation, which is consistent with our result that risk score is more powerful than IDH mutation after adjusting for some confounders in multivariate analysis (28). At the same time, LncRNAs have been reported to be dysregulated in considerable human diseases (such as cancer) (29). It has been increasingly evidenced that LncRNAs [such as immune-related (30) and autophagy-related (31) LncRNAs] exhibit diverse signaling transduction and regulatory functions, controlling the development and progression of the tumor.

In this study, a signature of glycolysis-related LncRNAs was developed and could be independently related to prognosis. Furthermore, two groups based on risk score were analyzed by various methods such as LASSO regression, Cox regression, ROC curve analysis, and GSEA. The mentioned signature was significantly related to OS and could be further considered to be a novel potential molecular therapeutic target.

In the signature, we identified six LncRNAs (FOXD2-AS1, AC062021.1, AF131216.5, LINC00844, CRNDE and LINC00665). FOXD2-AS1 (32, 33), LINC00844 (34, 35), CRNDE (5, 6), and LINC00665 (36, 37) were reported to have the similar biological functions and to be associated with prognosis, cell proliferation, invasion, cell cycle, and metastasis. Meanwhile, as demonstrated by existing reports, glycolysis activity has implicated in the cell proliferation (9–11), cytotoxic activity (38), immune response (39, 40), drug resistance (41, 42), poor prognosis (43–46), and tumor microenvironment (47) in most cancers. In accordance with functional enrichment analysis, we observed identified glycolysis-related LncRNAs were associated with cell cycle and cell proliferation. Thus, it is speculated that the mentioned LncRNAs may impact poor prognosis, cell proliferation, invasion, cell cycle, and metastasis through glycolytic biologic processes.

However, other glycolysis-related LncRNAs (such as AF131216.5 and AC062021.1) have not been reported yet. Basic biological experiments (*in vivo* or *in vitro*) and clinical researches are required to verify further the functional features. In addition, the whole six glycolysis-related LncRNAs, identified in the training cohort were not completely comprised either in The Cancer Genome Atlas database or in Gene Expression Omnibus database, so we had to select another cohort from the CGGA database as the validation cohort, which limited the generalizability of our conclusions to some extent.

## Conclusion

This study confirmed that glycolysis-related LncRNAs can impact poor prognosis, cell proliferation, invasion, cell cycle, and metastasis *via* glycolytic biologic processes, and the risk model based on glycolysis-related LncRNAs can significantly predict prognosis and may act as the therapeutic targets in the future.

## Data Availability Statement

Our study used public database, which can be found here: http://www.cgga.org.cn/.

## Ethics Statement

The present study was approved by the Institutional Review Board of the Beijing Tiantan Hospital affiliated with Capital Medical University.

## Author Contributions

YW, CZ, and WZ conceptualized and designed the study. YW, XG, XW, JP, PL, BM, PK, DL, and LY acquired the data. YW and WZ analyzed and interpreted the data. YW wrote and reviewed the manuscript. WJ and CZ supervised the study. All authors contributed to the article and approved the submitted version.

## Funding

This project was supported by the Beijing Municipal Health Commission of China (No. PXM2019_026280_000002), National Natural Science Foundation of China (No. 81802483 and No. 82071996), the Capital’s Funds for Health Improvement and Research (No. 2018-1-1071), and Beijing Hospitals Authority Youth Program (No. QML20190507)

## Conflict of Interest

The authors declare that the research was conducted in the absence of any commercial or financial relationships that could be construed as a potential conflict of interest.
