# Supplementary material for: Identification of a Glycolysis-Related LncRNA Signature to Predict Survival in Diffuse Glioma Patients
Source: Front Oncol. 2021 Feb 5;10:597877. doi: 10.3389/fonc.2020.597877 (PMC7892596; doi:10.3389/fonc.2020.597877)
Supplement: Supplementary file 1 [file DataSheet_1.pdf]

Supplementary Material

1. Supplementary Figure 1

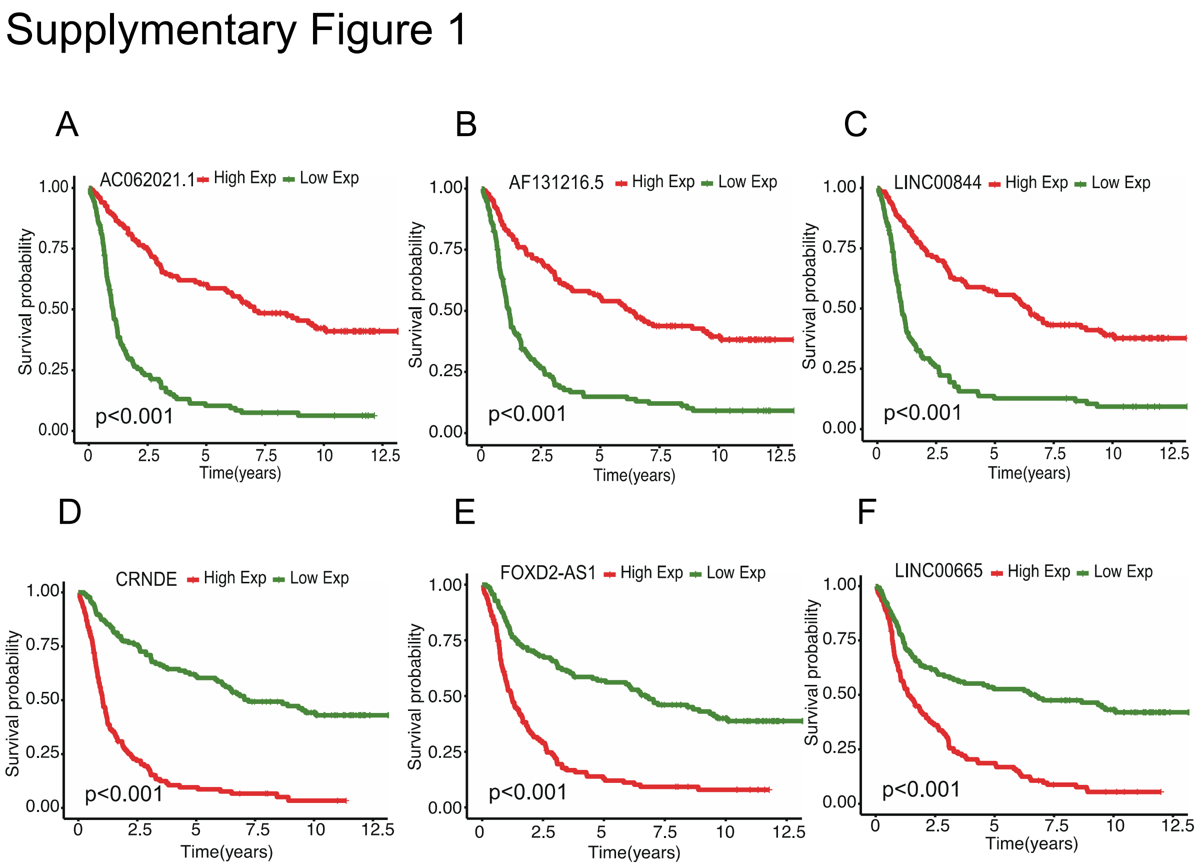

Supplementary Figure 1. Mentioned genes in Kaplan-Meier analysis in the validation cohort.

2. Supplementary Figure 2

Supplementary Figure 2

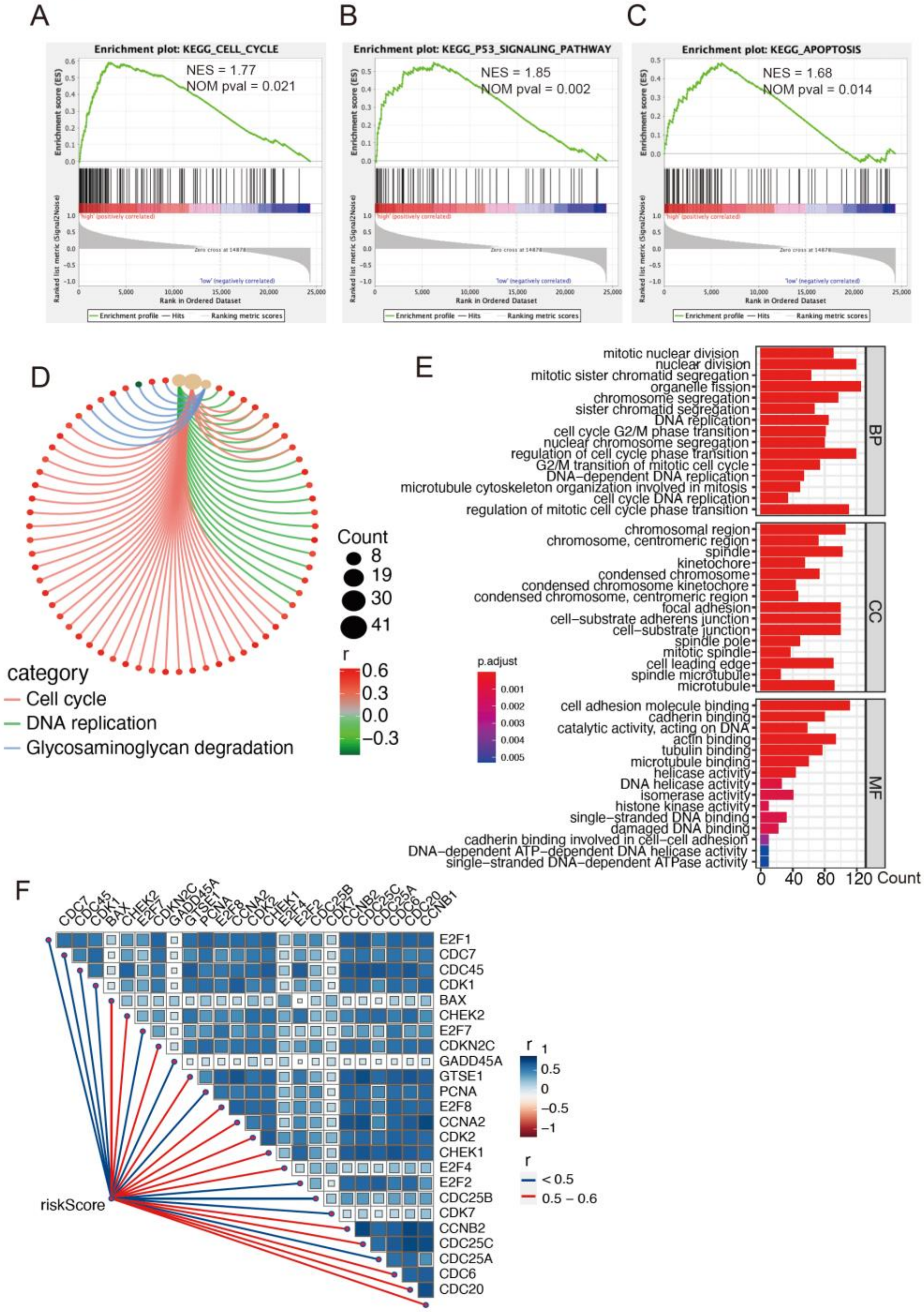

Supplementary Figure 2. Enrichment analysis for validation cohort. (A-C) GSEA enrichment between low-and high-risk groups revealing cell cycle and signaling pathway regulating cell proliferation. KEGG (D) and GO (E) analysis indicting the biological process risk score was mainly involved in cell proliferation. (F) The association of risk score with gene regulating cell cycle, all P value <0.001.
